# Disparities in COVID-19 Vaccination Status Among Long-Term Care Facility Residents — United States, October 31, 2022–May 7, 2023

**DOI:** 10.15585/mmwr.mm7240a4

**Published:** 2023-10-06

**Authors:** Emily Haanschoten, Heather Dubendris, Hannah E. Reses, Kira Barbre, Lu Meng, Andrea Benin, Jeneita M. Bell

**Affiliations:** ^1^Division of Healthcare Quality Promotion, National Center for Emerging and Zoonotic Infections, CDC; ^2^Lantana Consulting Group, East Thetford, Vermont; ^3^Goldbelt C6, Chesapeake, Virginia.

**Summary**

**What is already known about this topic?**

Long-term care (LTC) facility residents are vulnerable to SARS-CoV-2 infection because of their advanced age, medical complexity, and congregate living situation; vaccination is an effective means for reducing COVID-19 incidence in this population.

**What is added by this report?**

COVID-19 vaccination coverage among residents of participating LTC facilities within the National Healthcare Safety Network differed by race and geography. Bivalent COVID-19 vaccination rates were lowest among LTC facility residents in the South and Southeast U.S. regions and among Black or African American and multiracial residents.

**What are the implications for public health practice?**

Strategies aimed at increasing COVID-19 vaccination coverage should consider these demographic disparities to develop and implement **targeted** strategies to reduce inequities in COVID-19 morbidity and vaccination coverage.

## Abstract

Residents of long-term care (LTC) facilities constitute a population that is vulnerable to SARS-CoV-2 infection; COVID-19 vaccination effectively reduces severe COVID-19 in these settings. To examine demographic differences in primary and up-to-date vaccination status against COVID-19 among LTC facility residents, a descriptive analysis of COVID-19 vaccination data from the National Healthcare Safety Network (NHSN) COVID-19 vaccination data from October 31, 2022, to May 7, 2023, were analyzed. Being up to date was defined as having received a bivalent COVID-19 vaccine dose or having completed a primary vaccination series <2 months earlier. Geographic disparities in vaccination coverage were identified, with substantially lower prevalences of up-to-date status among LTC facility residents in the South (Region 6) (37.7%) and Southeast (Region 4) (36.5%) than among those in the Pacific Northwest (Region 10) (53.3%) and Mountain West (Region 8) (59.6%) U.S. Department of Health and Human Services regions. Up-to-date status was lowest among Black or African American (39.9%) and multiracial (42.2%) LTC facility residents. Strategies to increase up-to-date COVID-19 vaccination among LTC facility residents could include and address these geographic and racial differences.

## Introduction

Long-term care (LTC) facility residents are vulnerable to SARS-CoV-2 infection because of their often advanced age, medical complexity, and congregate setting (*1*). Vaccination against COVID-19 effectively reduces severe COVID-19 among persons living in these environments (*2*). Previous work has demonstrated racial and ethnic differences in COVID-19 vaccination coverage among the general population (*3*). This is the first examination of the National Healthcare Safety Network's demographic data among LTC facility residents for COVID-19 vaccination allowing for the exploration of vaccination coverage differences by race, age, gender, and geography. The purpose of this analysis is to examine demographic differences in primary and up-to-date vaccination status against COVID-19 among LTC facility residents. Findings from this analysis can be used to better understand heterogenous vaccination coverage and guide the development and implementation of strategies to increase up-to-date COVID-19 vaccination status among this population.

## Methods

In March 2022, NHSN* began optional, person-level surveillance of COVID-19 vaccination status among LTC facility residents in addition to ongoing weekly aggregated facility-level COVID-19 vaccination surveillance (*4*). COVID-19 vaccination data collection includes a weekly count of residents in LTCs with a stay of >24 hours by vaccination status. This study represents a retrospective, descriptive analysis of LTC facility resident data from LTC facilities that voluntarily reported person-level data to NHSN during October 31, 2022–May 7, 2023. These facilities included nursing homes, assisted living, and intermediate care facilities for persons with intellectual disabilities.

 Records from the most recent week of data submitted for each resident were included to prevent the inadvertent inclusion and analysis of duplicate records. Descriptive statistics were calculated for each demographic category by vaccination status, including completion of the primary COVID-19 vaccination series and up-to-date status. Being up to date was defined as having received a bivalent COVID-19 vaccine dose or having completed a primary vaccination series <2 months earlier.^†^ Department of Health and Human Services regions^§^ were used for geographical categorization. Statistical significance was assessed using chi-square tests of independence, with α = 0.05 considered statistically significant. All analyses were completed using SAS (version 9.4; SAS Institute). This activity was reviewed by CDC, deemed not research, and was conducted consistent with applicable federal law and CDC policy.^¶^

## Results

Among the 15,571 facilities enrolled in NHSN, records from 1,797 (11%) were eligible for inclusion, and >99% of records were reported by nursing homes (Table). COVID-19 vaccination varied substantially by LTC resident demographic characteristics. Up-to-date COVID-19 vaccination status was lower among residents of facilities in U.S. Department of Health and Human Services (HHS) Regions 4 (Alabama, Florida, Georgia, Kentucky, Mississippi, North Carolina, South Carolina, and Tennessee) (36.5%) and 6 (Arkansas, Louisiana, New Mexico, Oklahoma, and Texas) (37.7%) than among residents in HHS Region 10 (Alaska, Idaho, Oregon, and Washington) (53.3%) and 8 (Colorado, Montana, North Dakota, South Dakota, Utah, and Wyoming) (59.6%) (p<0.001). Although persons reporting American Indian, Asian, and Native Hawaiian or other Pacific Islander race represented <2% of the study population, the prevalences of up-to-date coverage among residents in these demographic groups were the highest overall (54.6%, 56.2%, and 60.6%, respectively), whereas coverage was lowest among residents who were Black or African American (39.9%) and multiracial (42.2%). A lower percentage of Hispanic or Latino (Hispanic) residents were up to date (36.5%) than were non-Hispanic residents (44.5%) (p<0.001). Up-to-date coverage increased with age: 46.0% of residents aged ≥75 years were up to date compared with 37.7% of residents aged 30–49 years. Up-to-date coverage was higher among female residents (44.6%) than among male residents (42.4%) (p<0.001).

**TABLE:** Characteristics of long-term care facility residents by COVID-19 vaccination status — National Healthcare Safety Network, United States, October 31, 2022–May 7, 2023

Characteristic	No. (%)			Mean no. of booster doses received (IQR)^†^	p-value^§^
	Total	Completed primary series	Up to date*		
**Facility type**					
Nursing home	**132,999 (99.2)**	111,631 (83.9)	57,998 (43.6)	1.5 (0–2)	<0.001
Assisted living	**943 (0.7)**	888 (94.2)	547 (58.0%)	2.0 (1–3)	
Intermediate care for persons with intellectual disabilities	**170 (0.1)**	163 (95.9)	147 (86.5%)	2.1 (2–3)	
**Age group, yrs**	**134,112**	112,682	58,692	—	—
Median (range)	**80.0 (0–122)**	80.0 (0–122)	81.0 (0–122)	—	—
0–29	**901 (0.7)**	626 (69.5)	288 (32.0)	0.9 (0–2)	<0.001
30–49	**2,886 (2.2)**	2,050 (71.0)	1,088 (37.7)	1.0 (0–2)	
50–64	**14,560 (10.9)**	11,201 (76.9)	5,673 (39.0)	1.2 (0–2)	
65–74	**28,148 (21.0)**	22,746 (80.8)	11,360 (40.4)	1.3 (0–2)	
≥75	**87,617 (65.3)**	76,059 (86.8)	40,283 (46.0)	1.5 (0–3)	
**Gender**					
Female	**81,474 (60.8)**	69,081 (84.8)	36,341 (44.6)	1.5 (0–2)	<0.001
Male	**52,246 (39.0)**	43,258 (82.8)	22,168 (42.4)	1.4 (0–2)	
Other	**378 (0.3)**	332 (87.8)	179 (47.4)	1.9 (1–3)	
**Ethnicity**					
Hispanic	**3,997 (3.0)**	3,244 (81.2)	1,457 (36.5)	1.2 (0–2)	<0.001
Not Hispanic	**116,752 (87.1)**	98,392 (84.3)	51,933 (44.5)	1.5 (0–2)	
Declined	**1,201 (0.9)**	971 (80.8)	450 (37.5)	1.2 (0–2)	
Unknown	**12,155 (9.0)**	10,050 (82.7)	4,848 (39.9)	1.4 (0–2)	
**Race**					
AI only	**747 (0.6)**	650 (87.0)	408 (54.6)	1.6 (1–3)	<0.001
Asian only	**1,309 (1.0)**	1,155 (88.2)	736 (56.2)	1.8 (1–3)	
Black or African American only	**13,912 (10.4)**	11,199 (80.5)	5,556 (39.9)	1.3 (0–2)	
NHOPI only	**251 (0.2)**	220 (87.6)	152 (60.6)	1.8 (1–3)	
White only	**106,214 (79.2)**	89,919 (84.7)	47,408 (44.6)	1.5 (0–3)	
Multiracial	**386 (0.3)**	302 (78.2)	163 (42.2)	1.3 (0–2)	
Unknown	9,919 (7.4)	8,129 (82.0)	3,785 (38.2)	1.4 (0–2)	
Declined	1,374 (1.0)	1,108 (80.6)	484 (35.2)	1.2 (0–2)	
**HHS region^¶^**					
1	**8,122 (6.1)**	7,305 (89.9)	4,317 (53.2)	1.7 (1–3)	<0.001
2	**5,609 (4.2)**	5,053 (90.1)	2,293 (40.9)	1.5 (1–2)	
3	**15,556 (11.6)**	13,431 (86.3)	7,482 (48.1)	1.6 (1–3)	
4	**41,993 (31.3)**	33,798 (80.5)	15,328 (36.5)	1.2 (0–2)	
5	**22,417 (16.7)**	19,078 (85.1)	10,732 (47.9)	1.6 (1–3)	
6	**12,909 (9.6)**	10,310 (79.9)	4,867 (37.7)	1.2 (0–2)	
7	**12,843 (9.6)**	11,263 (87.7)	6,507 (50.7)	1.7 (1–3)	
8	**4,415 (3.3)**	3,936 (89.2)	2,632 (59.6)	1.9 (1–3)	
9	**7,789 (5.8)**	6,380 (81.9)	3,223 (41.4)	1.4 (0–2)	
10	**2,459 (1.8)**	2,128 (86.5)	1,311 (53.3)	1.7 (1–3)	

**Abbreviations**: AI = American Indian; HHS = U.S. Department of Health and Human Services; NHOPI = Native Hawaiian or other Pacific Islander.

* Receipt of a bivalent booster dose or completion of the primary vaccination series <2 months earlier.

^†^ Booster doses available include both monovalent and bivalent vaccination for this period.

^§^ Chi-square test of independence for up-to-date vaccination.

^¶^ https://www.hhs.gov/about/agencies/regional-offices/index.html

## Discussion

Results from this analysis, the first to assess demographic and geographic disparities in up-to-date COVID-19 vaccination coverage among LTC facility residents using person-level data reported to NHSN, highlight geographic and racial and ethnic disparities in coverage among residents. These findings underscore the importance of improving the understanding of factors contributing to these geographic and demographic differences to guide public health practice and resource allocation (*5*). These findings are consistent with previously reported substantial population-level demographic disparities in COVID-19 vaccination coverage between Hispanic and non-Hispanic White populations (*6*). Low vaccination coverage and high levels of vaccine hesitancy have been found in the general population of the southeastern United States, mirroring the findings in this analysis (*7*). Barriers to accessing vaccination, including vaccination clinic availability and low vaccine confidence and demand, might contribute to low coverage and might account for some of the differences in vaccination coverage observed within the LTC facility resident population (*7*).

Future efforts are underway to encourage additional facilities to report person-level data, which would facilitate further analyses of demographic disparities, such as in vaccine effectiveness and potential associations with rates of infection. A recent publication reported that vaccine effectiveness was 31% among nursing home residents who were up to date with COVID-19 vaccination (*8*). This analysis was limited to aggregate data at the facility level and could not stratify by resident characteristics. Increased reporting of person-level data would facilitate a better understanding of the effect of COVID-19 vaccination in LTC settings.

### Limitations

The findings in this report are subject to at least two limitations. First, because person-level reporting is optional, only 11% of LTC facilities that report to NHSN were included in this analysis. Thus, the facilities reflected in this analysis might not be representative of all LTC facilities in a given region. Second, the categorization of residents as "unknown" based on self-reported ethnic or racial status could bias some vaccination coverage results. However, less than 10% of residents were categorized as “unknown” in this analysis. 

### Implications for Public Health Practice

Residents of LTC facilities should receive COVID-19 vaccination irrespective of their demographic characteristics to protect them from COVID-19 in congregate living environments. Most recent guidance indicates that persons who are aged ≥65 years or who are immunocompromised should consider additional bivalent vaccine doses. (*9*) Surveillance data reported to NHSN are an important tool to effectively monitor vaccination coverage among LTC facility residents as part of the COVID-19 public health response. As COVID-19 vaccination guidance evolves, strategic planning to increase vaccination coverage should include considerations to target and reduce demographic disparities.

## Footnotes

* CDC’s NHSN is the nation’s leading health care associated–infections surveillance system. COVID-19 vaccination surveillance data are reported to NHSN through COVID-19 Vaccination Modules. https://www.cdc.gov/nhsn/ltc/weekly-covid-vac/index.html

†
www.cdc.gov/nhsn/pdfs/hps/covidvax/UpToDateGuidance-508.pdf

§
https://www.hhs.gov/about/agencies/regional-offices/index.html

¶ 45 C.F.R. part 46.102(l)(2), 21 C.F.R. part 56; 42 U.S.C. Sect. 241(d); 5 U.S.C. Sect. 552a; 44 U.S.C. Sect. 3501 et seq.
